# Formant analysis in dysphonic patients and automatic Arabic digit speech recognition

**DOI:** 10.1186/1475-925X-10-41

**Published:** 2011-05-30

**Authors:** Ghulam Muhammad, Tamer A Mesallam, Khalid H Malki, Mohamed Farahat, Mansour Alsulaiman, Manal Bukhari

**Affiliations:** 1 Computer Engineering Department, College of Computer and Information Sciences, King Saud University, Riyadh, Saudi Arabia; 2 ENT Depatment, College of Medicine, King Saud University, Riyadh, Saudi Arabia; 3 Otolaryngology Department, College of Medicine, Al-Menoufiya University, Egypt

**Keywords:** speech recognition, voice disorders, formants, Arabic digits

## Abstract

**Background and objective:**

There has been a growing interest in objective assessment of speech in dysphonic patients for the classification of the type and severity of voice pathologies using automatic speech recognition (ASR). The aim of this work was to study the accuracy of the conventional ASR system (with Mel frequency cepstral coefficients (MFCCs) based front end and hidden Markov model (HMM) based back end) in recognizing the speech characteristics of people with pathological voice.

**Materials and methods:**

The speech samples of 62 dysphonic patients with six different types of voice disorders and 50 normal subjects were analyzed. The Arabic spoken digits were taken as an input. The distribution of the first four formants of the vowel /a/ was extracted to examine deviation of the formants from normal.

**Results:**

There was 100% recognition accuracy obtained for Arabic digits spoken by normal speakers. However, there was a significant loss of accuracy in the classifications while spoken by voice disordered subjects. Moreover, no significant improvement in ASR performance was achieved after assessing a subset of the individuals with disordered voices who underwent treatment.

**Conclusion:**

The results of this study revealed that the current ASR technique is not a reliable tool in recognizing the speech of dysphonic patients.

## Introduction

Among the tasks for which machines may simulate human behavior, automatic speech recognition (ASR) has been foremost since the advent of computers. A device to understand speech, however, needs a calculating machine capable of making complex decisions, and, practically, one that could function as rapidly as humans. As a result, ASR has grown rapidly in proportion to other areas of pattern recognition (PR) based in a large part on the power of computers to capture a relevant signal and transform it into pertinent information, i.e., recognizing patterns in the speech signal [1].

There has been a growing interest in objective assessment of acoustic variables in dysphonic patients in recent years. Voice pathology detection and classification is a topic which has interested the international voice community [2]. Most of the work in this field is concentrated on automatically diagnosing the pathology using digital signal processing methods [3-6]. For example, in the study of Dibazar et al, [3] five different vocal pathologies were detected using MFCC and fundamental frequencies. In their study, the highest recognition sensitivity was achieved with vocal fold paralysis while the lowest sensitivity was for hyperfunctional voice disorders.

In another study by Dubuisson et al [4], discrimination of normal and pathological voices was analyzed using correlation between different types of acoustic descriptors. Such descriptors were of two types; temporal and cepstral. Temporal descriptors included energy, mean, standard deviation, and zero crossing, while spectral descriptors included delta, mean, several moments, spectral decrease, roll-off, etc. It has been found that using spectral decrease and first spectral tri-stimulus in the Bark scale, and their correlation leads to correct classification rate between normal and pathological voices of 94.7% for pathological voices and 89.5% for normal ones with sustained vowels. These rates mean that 94.7% of the pathological voices were classified as pathological voices and 89.5% of the normal voices were classified as normal voices. The reason behind the higher rates for pathological voices is that the authors use features inspired from voice pathology assessment and the number of normal voice samples is much lower than that of pathological samples. The performance of linear predictive coding (LPC)-based spectral analysis to discriminate pathological voices of speakers affected by vocal fold edema was evaluated in the study of Costa et al [5]. Their results show that LPC-based cepstral method is a good way to represent changes in vocal tract by vocal fold edema. In another study, estimation of glottal noise from voice signals using short-term cepstral was used to discriminate pathological voices from normal voices [6]. It was found that glottal noise estimation correlated less with jitter and shimmer for pathologic voices and not significantly for normal voices. Miyamoto et al [7] investigated pose-robust audio-visual speech recognition of a person with articulation disorders resulting from cerebral palsy. They used multiple acoustic frames (MAF) as an acoustic feature and active appearance model (AAM) as a visual feature in their system. Their proposed audio-visual method resulted in an improvement of 7.3% in the word recognition rate at 5 dB signal-to-noise ratio compared to the audio-only method.

All of the above-mentioned studies used only sustained vowel /a/ as an input. Comparative evaluation between sustained vowel and continuous speech for acoustically discriminating pathological voices was studied by Parsa et al [8]. It was found in their experiment that classification of voice pathology was easier for sustained vowel than for continuous speech. On the other hand, automated intelligibility assessment was performed with context dependent phonological features using 50 consonant-vowel-consonant (CVC) words from six different types of voice disordered speakers in the study of Middag et al [9]. Their evaluation revealed that the root mean squared error of the discrepancies between perceived and computed intelligibilities can be as low as 8 on a scale of 0 to 100. Automatic recognition of Polish words was carried out in the study Wielgat et al [10], where the input was speech from voice disordered Polish children. They used MFCC and human factor cepstral coefficients (HFCC) to recognize words with confusing phonemes. In their experiment, HFCC performed better than MFCC. In a recent work, automatic recognition system evaluated speech disorders in head and neck cancer, where the speakers were German natives [11]. Intelligibility was quantified by speech recognition on recordings of a standard text read by laryngectomized patients with cancer of the larynx or hypopharynx and patients who had suffered from oral cancer. Both patient groups showed significantly lower word recognition rates than an age-matched control group.

In the current study, a conventional ASR system was used for evaluation of six different types of voice disordered patients speaking Arabic digits. MFCC and GMM (Gaussian mixture model)/HMM (hidden Markov model) were used as features and classifier, respectively. The recognition results were analyzed for types of diseases. Effects on performance before and after clinical management in a subset of the disordered voices were also investigated. Finally, the first four formants (F1, F2, F3, and F4) of vowel /a/ present in the digits were extracted to make a comparison of distortion in terms of formants for different voice disorders. We believe that this is the first such work that tries to examine the accuracy of ASR in Arabic speech of people with pathological voices. Also the comparison of ASR performance between pre and post management (surgical or medical) may provide additional interest to other language communities now investigating ASR as a mean of examining outcomes of treatments.

## Materials and methods

### Data

The study has been approved by the ethical committee, Faculty of Medicine, King Saud University. The medical records have been reviewed to extract recorded speech samples from those patients with different voice disorders. In addition, speech samples were recorded for 50 normal subjects with no previous or current history of voice disorders. All speakers were native Arabs with age range from 18 to 50 years. Two sets of data were used: one for training the ASR system and the other for testing. For training, speech samples from speakers who have no voice disorders (normal speakers) were used. An in-house database was created from ten Arabic digits (1 to 10) for training [12]. A total of fifty speakers uttered the ten Arabic digits with ten repetitions. The first 40 speakers were used in training, while the rest 10 speakers were reserved for testing. The sampling rate was set to 16 kHz. Table 1 lists the ten Arabic digits that were used in the experiment.

**Table 1:** Arabic digits used in the study

Symbol	Digits	Arabic writing	Syllables	Number of syllables
**D1**	**Wahed**	واحد	CV-CVC	**2**
**D2**	**Athnayn**	أثنين	CVC-CVCC	**2**
**D3**	**Thalathah**	ثلاثة	CV-CV-CVC	**3**
**D4**	**Arbaah**	أربعة	CVC-CV-CVC	**3**
**D5**	**Khamsah**	خمسة	CVC-CVC	**2**
**D6**	**Setah**	ستة	CVC-CVC	**2**
**D7**	**Sabaah**	سبعة	CVC-CVC	**2**
**D8**	**Thamanyah**	ثمانية	CV-CV-CV-CVC	**4**
**D9**	**Tesaah**	تسعة	CVC-CVC	**2**
**D10**	**Ashra**	عشرة	CVC-CVC	**2**

For test data, total 62 speakers of six different types of voice disorders gave their Arabic digit speech. For each type of voice disorders we had at least 10 speakers. The speakers' age ranged from 18 to 50 years, and all of them were native Arabs. Test data samples were recorded in different sessions at the Research Chair of Voice and Swallowing Disorders, King Abdul Aziz University Hospital, King Saud University, Riyadh by experienced phoniaticians in a sound treated booth using a standardized recording protocol. All the patients' speech samples were recorded using the KayPentax computerized speech laboratory (CSL Model 4300). The patients were asked to count the Arabic digits from 1-10 and the sampling rate was set to 16 kHz. Table 2 lists details of speech data used for training and testing.

**Table 2:** Details of speech samples used for training and testing

Training			Testing		
**Disorder**	**Number of speakers**		**Disorder**	**Number of speakers**	
			
	**Male**	**Female**		**Male**	**Female**

**Normal**	**20**	**20**	**Normal**	**5**	**5**

			Cyst	6	5
			
			LPRD	6	5
			
			SD	6	4
			
			Sulcus	6	5
			
			Nodules	5	4
			
			Polyp	5	5

40 normal (without voicing disorder) speakers, with equal number of male and female, were used in training. Five normal male and female speakers each and 62 speakers from six different voicing disorders were used in testing.

The six types of voicing disorders considered in this work were vocal fold cysts, laryngopharyngeal reflux disease (LPRD), spasmodic dysphonia (SD), sulcus vocalis, vocal fold nodules, and vocal fold polyps. A brief description of these disorders and their effects on voice is given below [13-17].

**Figure 1:**
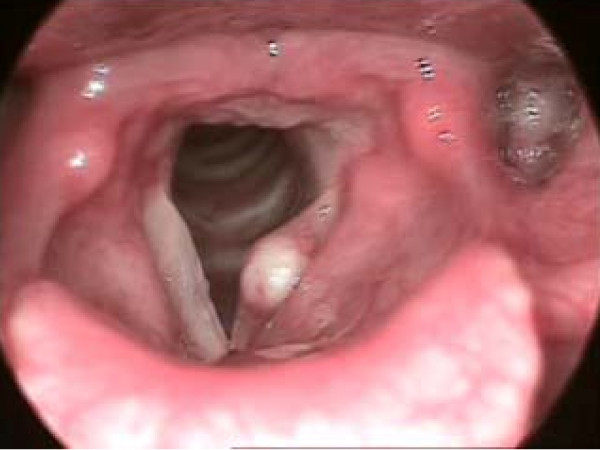
**Left vocal fold cyst**. There is a presence of cyst in the left vocal fold. Vocal fold cysts are subepidermal epithelial-lined sacs located within the lamina propria, and may be mucus retention or epidermoid in origin. The voice often sounds diplophonic (particularly with epidermoid cysts), whereby there is great pitch instability.

**Figure 2:**
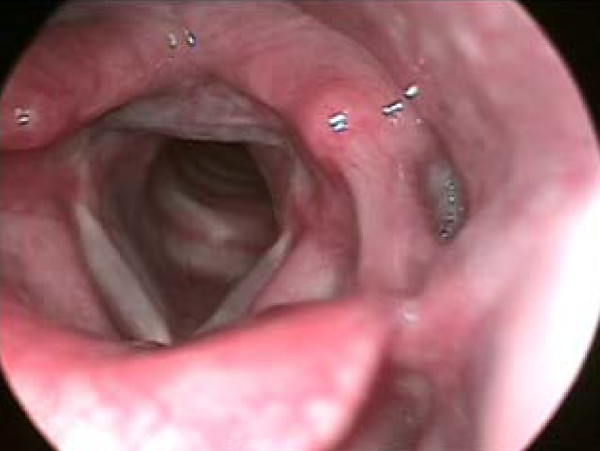
**Laryngopharyngeal reflux disease (LPRD)**. The patient suffers from LPRD. LPRD is the retrograde movement of gastric contents into the laryngopharynx leading to symptoms referable to the larynx, hypopharynx, and nasopharynx. This disease causes abnormal vocal cord vibration.

**Figure 3:**
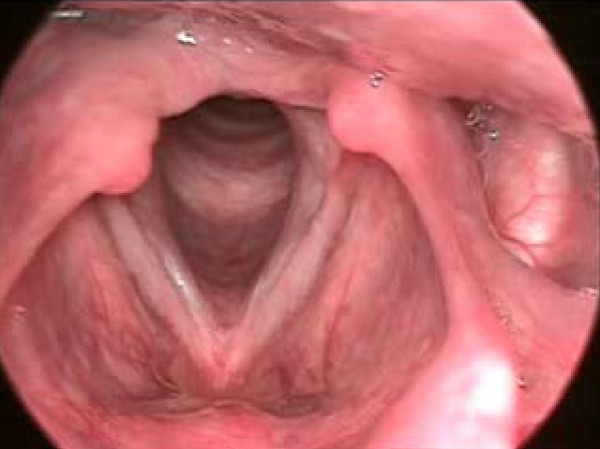
**Sulcus vocalis**. Sulcus vocalis is a linear depression on the mucosal cover of the vocal folds, parallel to the free border. It is of variable depth, and usually bilateral and symmetrical. It inhibits complete closure of the vocal folds and causes stiffness in the vocal fold mucosa. In the figure sulcus vocalies can be seen on the right hand side.

**Figure 4:**
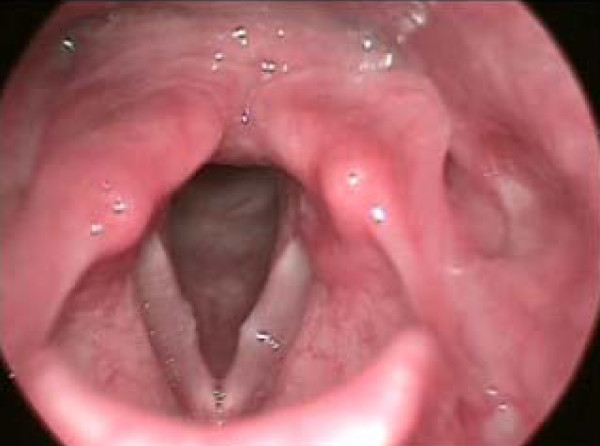
**Vocal fold nodules**. Vocal fold nodules are defined as bilateral symmetric epithelial swelling of the anterior/mid third of the true vocal folds. Vocal fold nodules frequently interfere with vocal fold closure, so dysphonia is a common symptom. In the figure vocal fold nodule can be seen on the right hand side.

**Figure 5:**
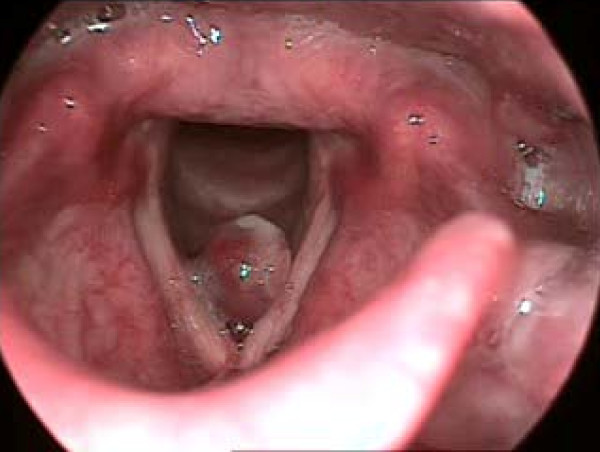
**Right vocal fold polyp**. Vocal fold polyps are usually unilateral, occasionally pedunculated masses encountered on the true vocal fold. Polyps result in excess air egress during phonation, and are associated with earlier vocal fatigue, frequent voice breaks in singers, and worsening dysphonia.

(a) Vocal fold cysts are subepidermal epithelial-lined sacs located within the lamina propria, and may be mucus retention or epidermoid in origin. The voice often sounds diplophonic (particularly with epidermoid cysts), whereby there is great pitch instability (Figure 1).

(b) LPRD is the retrograde movement of gastric contents (acid and enzymes, such as pepsin) into the laryngopharynx leading to symptoms referable to the larynx, hypopharynx, and nasopharynx. Symptoms include dysphonia, globus pharyngeus, mild dysphagia, chronic cough, excessive throat mucus, chronic throat clearing, etc (Figure 2).

(c) Spasmodic dysphonia (SD) is a neuromuscular disorder. It is characterized by strained, strangled, and interrupted voice, with pitch and phonatory breaks and difficulty coordinating respiration with phonation.

(d) Sulcus vocalis is a linear depression on the mucosal cover of the vocal folds, parallel to the free border. It is of variable depth, and usually bilateral and symmetrical. It inhibits complete closure of the vocal folds and causes stiffness in the vocal fold mucosa (Figure 3).

(e) Vocal fold nodules are defined as bilateral symmetric epithelial swelling of the anterior/mid third of the true vocal folds. These are seen in children, adolescents, and female adults working in professions with high voice demands. Vocal fold nodules frequently interfere with vocal fold closure, so dysphonia is a common symptom (Figure 4).

(f) Vocal fold polyps are usually unilateral, occasionally pedunculated masses encountered on the true vocal fold. They occur more often in males, and they often occur after intense intermittent voice abuse. Polyps result in excess air egress during phonation, and are associated with earlier vocal fatigue, frequent voice breaks in singers, and worsening dysphonia (Figure 5).

**Figure 6:**
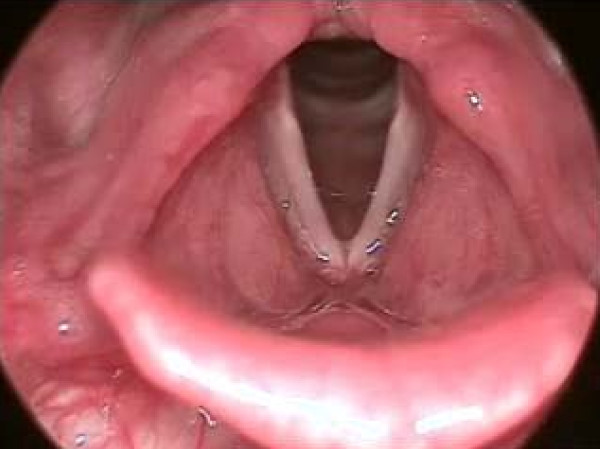
**Normal larynx**. for comparison of vocal tracts between different voicing disorder and normal larynx, Figure 6 shows picture of a normal larynx.

A picture of normal larynx is shown in Figure 6.

### Experiments with ASR system

The experiment in this work was conducted on a connected phoneme task constituting isolated Arabic digits. Each phoneme was modeled by a three state HMM. The state transition was left-to-right. Observation probability density functions were modeled using GMM. The number of mixtures in the model of each state was varied between 1, 4, 8, and 16. All training and recognition experiments were implemented with the HTK package [18]. Training was performed using normal speech, while testing was performed using normal and voice disordered speech.

The parameters of the system were: 16 kHz sampling rate with a 16 bit sample resolution, 25 milliseconds Hamming window with a step size of 10 milliseconds, and the pre-emphasis coefficient was 0.97. As features, 12 MFCC and 24 MFCC (12 MFCC plus their delta coefficients) were used.

A second experiment was also carried out where pre- and post- management samples of 16 voice disordered patients who underwent treatments were compared in order to see whether there was any improvement in ASR after the management. Twelve patients received surgical intervention (2 having vocal fold cyst, 5 with spasmodic dysphonia, and 5 with sulcus vocalis) and four patients were medically treated (LPRD).

Also, a formant-based analysis of the Arabic vowel /a/ was carried out for different types of voice disordered speech. First four formants of the vowel /a/ present in Arabic digits were analyzed. This vowel is present in all of the ten Arabic digits (Table 1). Three frames in the middle of vowel /a/ in each utterance were considered to minimize the co-articulation effects. Middle frames are manually detected. Formant values of these frames are averaged to determine final four formants. Praat software [19] was used for voice analysis of samples in this study.

## Results and discussion

**Figure 7:**
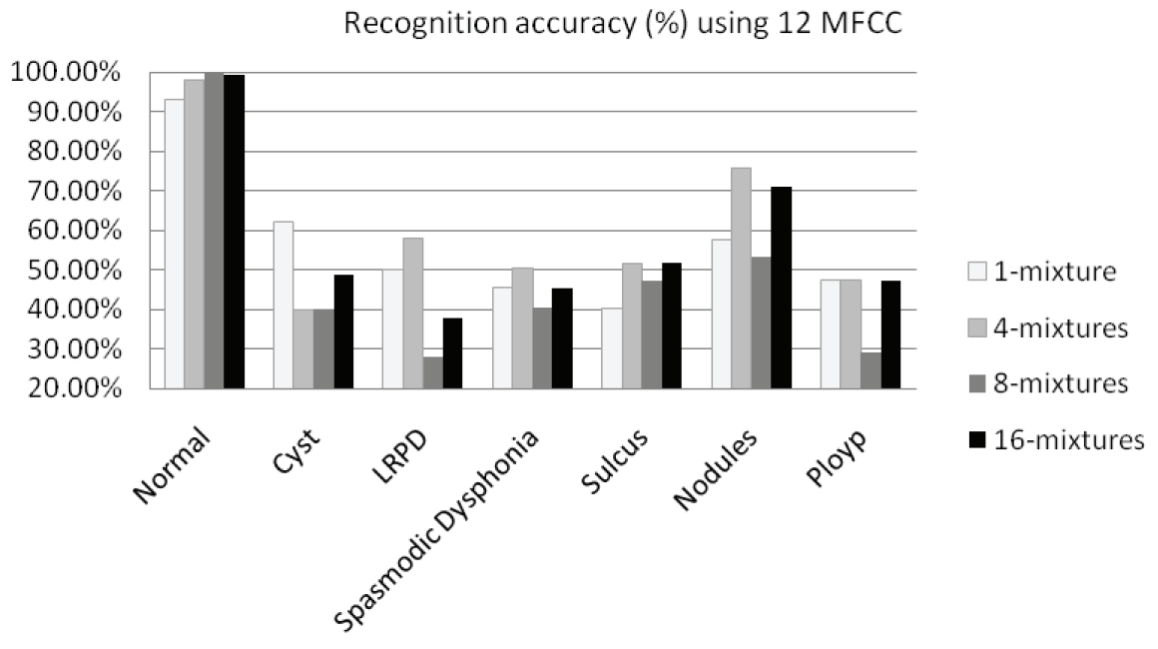
**Recognition accuracy (%) of different voicing conditions using 12 MFCC features**. The figure shows comparison of the performance of Arabic digit recognition system using 12 MFCC features for normal voice and six other voicing disorders. For all Gaussian mixtures, normal voice performs the best, which is quite usual. Among the voicing disorder, nodules have the highest performance, which is 76% using four mixtures. The lowest performance is with polyp. The number of mixture has no specific contribution towards the recognition performance for disordered voice.

**Figure 8:**
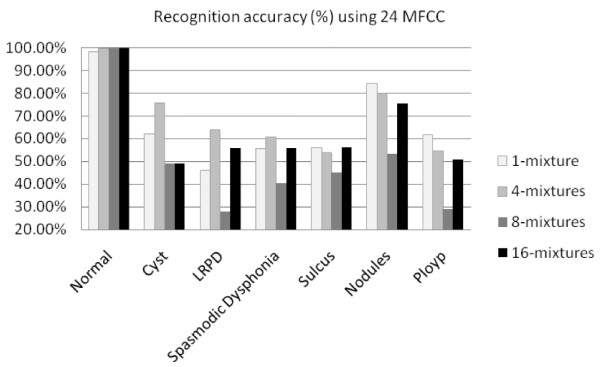
**Recognition accuracy (%) of different voicing conditions using 24 MFCC features**. The figure shows comparison of the performance of Arabic digit recognition system using 24 MFCC features for normal voice and six other voicing disorders. For all Gaussian mixtures, normal voice performs the best, which is quite usual. The accuracy reaches to 100% for higher mixtures. Among the voicing disorder, nodules have the highest performance, which is 84.5% using one mixture. The lowest performance is with sulcus. Again, the number of mixture has no specific contribution towards the recognition performance for disordered voice.

Figures 7 and 8 show the recognition accuracy (%) for each type of voice disorders using 12 MFCC and 24 MFCC, respectively. Table 3 shows best accuracy obtained in different variables. From Figures 7 and 8, and Table 3, it can be seen that 100% recognition accuracy was obtained for Arabic digits spoken by normal speakers. There was a significant loss of accuracy on speech recognition of voice disordered samples. For example, Arabic digits spoken by patients with vocal folds polyps had recognition accuracy of 47% using 12 MFCC and 4 mixtures, and 61% using 24 MFCC and 1 mixture. Among the six different types of voice disorders, the least accuracy was obtained for sulcus vocalis (maximum accuracy of 56%) and the best accuracy was for vocal folds nodules (maximum accuracy of 84.50%). The least accuracy was obtained in sulcus vocalis because in such kind of disorders, the dysphonia or the change in the voice character is expected to be more severe than that in other lesions including nodules. This could be explained by the markedly impaired mucosal vibration of the vocal folds in sulcus vocalis due to affection of almost the whole layers of the mucosa (lamina propria) as the sulcus could extend deeply to involve the vocal ligament [20]. On the other hand, in other kinds of vocal folds lesions the pathology is mainly located in the superficial layer of lamina propria (superficial layer of the mucosa). Accordingly, the impairment of mucosal vibration will be less in superficial lesions than in sulcus vocalis which is subsequently reflected on the severity of voice impairment.

**Table 3:** Best accuracy (%) obtained in different voice disorders groups

Type of voice disorder	Best mixture	Best feature	Best accuracy
**Normal**	8 or 16	24	100%
**Cyst**	4	24	75%
**LPRD**	4	24	64%
**Spasmodic Dysphonia**	4	24	60%
**Sulcus**	1 or 16	24	56%
**Nodules**	1	24	84.50%
**Polyp**	1	24	61%

The best feature is 24 MFCC; however the best mixture varies with the types of disorder. The ASR system can correctly recognize all the digits spoken by persons with no voicing disorder. The performances with LPRD, spasmodic dysphonia, sulcus, and polyp are below 65% with the current ASR system.

In terms of performance of mixtures, it was found that there was no pattern of mixture for the best performance. In some of the disorders, the best performance was achieved with mixture 1, some with mixture 4, and some with mixture 8 and 16. In all of the disorders, 24 MFCC performed better than 12 MFCC. This indicates that adding time derivatives to the feature can improve the performance with voice disorders, as it can do with normal voice.

The recognition performances of Arabic digits ASR with pre- and post-management are shown in Table 4. In this table, only the best performances were shown. The table indicates that there was only little improvement in ASR performance achieved after management. For example, medical treatment for LPRD patients improved ASR performance from 62% to only 65%. This can be attributed to that, although management of these voice disorders could improve the patient's voice from the clinical point of view, it failed to significantly improve ASR performance.

**Table 4:** Best accuracy (%) obtained in pre- and post-management of patients

Type of voice disorder	Pre - management	Post-management
**Cyst**	72%	75%
**LPRD**	62%	65%
**Spasmodic Dysphonia**	59%	62%
**Sulcus**	54%	60%

The table indicates that there is only little improvement in ASR performance achieved after management. For example, medical treatment for Spasmodic Dysphonia patients improved ASR performance from 59% to only 62%. This can be attributed to that, although management of these voice disorders could improve the patient's voice from the clinical point of view, it failed to significantly improve ASR performance.

### Formant analysis of/a/for different types of voicing disorders

In Arabic, there are six vowels:/a/,/i/,/u/and their longer counterparts/a:/,/i:/,/u:/. Some researchers consider Arabic vowels to be eight in total, by adding two diphthongs as vowels, and this is normally considered to be the case for modern standard Arabic (MSA) [21]. By changing the vocal tract shape, different resonating frequencies are produced. Each of the preferred resonating frequencies of the vocal tract (corresponding to the relevant bump in the frequency response curve) is known as a formant. These are usually referred to as F1 indicating the first formant, F2 indicating the second formant, F3 indicating the third formant, etc. [22].

Table 5 shows the first four formants (F1, F2, F3, and F4) of the vowel /a/ in different voicing conditions. All the formants listed are in Hz. The F1 and F2 values of  /a/ sound in normal speech obtained in this study differ slightly from the values reported in other studies [22,23]. In the study of Alotaibi and Husain [22], F1 and F2 values for /a/ were (591, 1102), while in a second study by Newman and Verhoeven [23] those values were (695, 1590). The lower the F1 is, the closer the tongue is to the roof of the mouth. The value of F2 is proportional to the frontness or backness of the highest part of the tongue during the production of the vowel. The slight difference of formant frequencies between different studies comes from the fact that different sets of speakers are used in the studies, and formant frequencies for a specific vowel have a dependency on speakers' vocal tract length and vocal cavity size.

**Table 5:** Comparison between the first four formants of the vowel/a/in the tested Arabic digits in different voicing disorders

Voice Disorders	F1 (Hz)			F2 (Hz)			F3 (Hz)			F4 (Hz)		
	
	Mean	SD	P Value (t test)	Mean	SD	P Value (t test)	Mean	SD	P Value (t test)	Mean	SD	P Value (t test)
**Normal**	710	4		1492	53		2467	16		3647	45	

**Cyst**	528	107	0.001 >	1145	107	0.01	2452	115	0.001 >	4001	115	0.05

**LPRD**	747	107	0.03	1345	107	0.02	2398	115	0.001 >	3668	115	0.01

**Spasmodic dysphonia**	582	201	0.001 >	1178	101	0.01	2687	132	0.002	3536	132	0.04

**Sulcus**	658	324	0.04	1202	108	0.02	2512	439	0.03	3532	118	0.001 >

**Nodules**	612	104	0.001 >	1301	75	0.005	2598	360	0.02	3899	166	0.04

**Polyp**	821	377	0.01	1431	28	0.001 >	2880	250	0.005	3892	83	0.001 >

The speech obtained from all voice disordered patients had significantly (P < 0.05, Student's t-test) deviated formant values from that of normal. For instance, vocal fold cysts patients had F1 of 528 Hz, which has 107 Hz standard deviation (SD) from F1 of normal subjects. Similarly, F2 of the same disorder (1145 Hz) has SD of 107 Hz from that of normal. F1 value deviated most from normal in vocal fold cysts, followed by spasmodic dysphonia. The same was true for F2 value. F3 that corresponds to phoneme quality had the highest deviation from the normal in vocal fold polyps group, while F4 that corresponds to voice quality deviated most in vocal fold cysts.

The speech obtained from all voice disordered patients had significantly (P < 0.05, Student's t-test) deviated formant values from that of normal. For instance, vocal fold cysts patients had F1 of 528 Hz, which was 182 Hz less than F1 of normal subjects. Similarly, F2 of the same disorder (1145 Hz) was 347 Hz less than that of normal. F1 value deviated most from normal in vocal fold cysts, followed by spasmodic dysphonia. The same was true for F2 value. F3 that corresponds to phoneme quality had the highest deviation from the normal in vocal fold polyps group, while F4 that corresponds to voice quality deviated most in vocal fold cysts. This information can be embedded in ASR system to correctly recognize the type of the voice disorder from a sample of pathological voice.

The formant values were not consistent even in the same type of voice disorder. It varied between different samples and sometimes within the same sample. Sustained vowel speech sounds of voice disordered people exhibit a large range of behavior. The behavior can be characterized as nearly periodic or regular vibration, aperiodic or irregular vibration and sounds with no apparent vibration at all. All can be accompanied by varying degrees of noise which can be described as "breathiness". Voice disorders therefore commonly exhibit two characteristic phenomena: increased vibrational aperiodicity and increased breathiness compared to normal voices [6].

From Table 5, it was shown that F1 in sulcus vocalis patients had standard deviation of 324 and vocal fold polyp patients had that of 377. The high standard deviation indicates unstable nature of formants in each type of voice disorders. For every formant value in each type of disorder, the standard deviation was more than 100 with the exception of F2 in nodules and polyp cases. This is one of the reasons of low recognition accuracy of Arabic digits uttered by voice disordered patients. However, we find a relation between recognition accuracy and standard deviation of first two formants, which are more significant than F3 and F4 in ASR, of /a/ for different types of disorder. For example, sulcus and polyp have higher standard deviations (324 and 377, respectively) with F1 and lower recognition accuracies (less than 62%); nodules have lower standard deviation (104) and higher recognition accuracy (84%). With F2, sulcus has the highest standard deviation (108) and nodules has one of the lowest (75) ones.

Formants of the vowels can be studied further to embed it in feature extraction module of Arabic ASR designed for pathological voices. It can be mentioned that every word and syllable in Arabic language must contain at least one vowel. This analysis is expected to be helpful in future Arabic speech processing tasks such as vowel and speech recognition and classification of voice disorders.

## Conclusion

Arabic digits ASR performance in six different voice disorders was evaluated. Recognition accuracy varied between 56% and 82.50% in the disordered groups, while it was 100% in normal subjects. Performance was also checked in post-management condition, where there was no significant increase in recognition accuracy. In addition, the first four formants of /a/ sound in Arabic digits were analyzed in these voice disordered conditions. The formant values varied significantly across and within voice disorders groups. The results of this study revealed that the current ASR technique is far from reliability in recognizing the speech of dysphonic patients. More studies are needed to look for meaningful features in order to improve ASR performance in speech of pathological voices. Our future work includes evaluating the performance of the ASR system by (i) using acoustic models which are trained by speech samples of disorders, (ii) comparing different sets of feature vectors, and (iii) selecting optimal features.

## Competing interests

The authors declare that they have no competing interests.

## Authors' contributions

GM carried out the automatic speech recognition test, participated in the formant analysis, and drafted the manuscript. TAM carried out the formant analysis of the vowels and coordinated the study between King Abdul Aziz University Hospital and department of computer engineering. KHM conceived of the study and participated in its design. MF developed the database and performed the statistical analysis. MA participated in the automatic speech recognition test by setting up and fine tuning the parameters. MB helped to draft the manuscript and participated in the design of the study. All authors read and approved the final manuscript.
